# Influence of Hall Current and Viscous Dissipation on Pressure Driven Flow of Pseudoplastic Fluid with Heat Generation: A Mathematical Study

**DOI:** 10.1371/journal.pone.0129588

**Published:** 2015-06-17

**Authors:** Saima Noreen, Muhammad Qasim

**Affiliations:** Department of Mathematics, COMSATS Institute of Information Technology, Park Road, Chak Shehzad, Islamabad 44000, Pakistan; North China Electric Power University, CHINA

## Abstract

In this paper, we study the influence of heat sink (or source) on the peristaltic motion of pseudoplastic fluid in the presence of Hall current, where channel walls are non-conducting in nature. Flow analysis has been carried out under the approximations of a low Reynolds number and long wavelength. Coupled equations are solved using shooting method for numerical solution for the axial velocity function, temperature and pressure gradient distributions. We analyze the influence of various interesting parameters on flow quantities. The present study can be considered as a mathematical presentation of the dynamics of physiological organs with stones.

## 1 Introduction

The peristaltic transport of fluid in a tube or channel is of fundamental importance and occurs in chyme motion in gastrointestinal tract, urine transport from kidney to bladder, spermatozoa transport in human reproductive tract, heart lung machine, vasomotion of small blood vessels, sanitary and corrosive fluid transport etc. A mathematical formulation of physiological fluid flows provides a better understanding of the specific flow being modeled. The mechanism of flow can be better explained by comparing mathematical results and the experimental clinical data. An accurate mathematical study can serve as a mile stone to many peristaltic flows occurring in the human body. The peristaltic mechanism of viscous fluid was first studied by Latham [1] experimentally. Shapiro et al. [2], analyzed the peristaltic transport of viscous fluid using long wavelength and a low Reynolds number. Later, several others made useful investigations regarding the peristaltic transport of viscous fluid along with taking into account different geometries and assumptions of small Reynolds number, large wavelength, small/arbitrary amplitude ratio and small wave number.

A part from this, the non-Newtonian fluids are frequently encountered in food mixing, chyme movement in the intestine, blood flow at low shear rate, the flow of nuclear slurries, liquid metals and alloys. There are also situations where magnetic field characteristics in non-Newtonian fluids are significant. For instance, flow of mercury amalgams and lubrication with heavy oil and grease [3–9].

Current advances in the subject of Hall current involve Hall accelerators, nuclear power reactors, flight MHD and MHD generators. The Hall current IC switch or sensors are extremely useful to detects the presence or absence of a magnetic field and gives a digital signal for on and off. Large values of Hall current parameter in the presence of heavy-duty magnetic fields corresponds to Hall current, which is how, it shakes the current density and allows one to understand the impact of Hall current on the flow [10].

Hayat et al. [11] studied effects of Hall current on peristaltic flow of a Maxwell fluid in a porous medium. The effects of Hall current and heat transfer on MHD flow of a Burgers’ fluid under the pull of eccentric rotating disks has been examined by Siddiqui et. al [12]. In another paper Gad [13], examined the effect of Hall currents on interaction of pulsatile and peristaltic transport induced flows for a particle-fluid suspension.

Recently, studies [14–22] of heat and mass transfer in peristalsis have been considered by some researchers due to its applications in biomedical sciences. Heat transfer involves many complicated processes such as evaluating skin burns, destruction of undesirable cancer tissues, dilution technique in examining blood flow, paper making, food processing, vasodilation, metabolic heat generation and radiation between surface and its environment, metabolic heat generation and radiation between surface and its environment. It is clear from reviewing the existing literature, that no much attention has been given to the peristaltic flows with heat generation and Hall Currents, especially such attempts being further narrowed down for the case of non-Newtonian fluids.

To the best of authors knowledge no study is available that deals with Hall current effects on peristaltic transport of pseudoplastic fluid in presence of mixed convection and heat generation. Hence, relevant problem is modeled in wave frame of reference. The paper is structured as follows: Second section consists of mathematical modelling of fundamental equations. Discussion to plots is assigned in section three and the conclusions follow in section four.

## 2 Mathematical Modeling

We consider pseudoplastic fluid in an asymmetric channel of thickness *d*_1_ + *d*_2_. A sinusoidal wave of velocity *c* propagates along the non-conducting channel walls. Cartesian coordinates system $\left(\bar{X},\bar{Y}\right)$ has been considered with $\bar{X}$ parallel and $\bar{Y}$ perpendicular to the direction of wave movement. Moreover, constant magnetic field of strength *B*_0_ acts in the y—direction. The induced magnetic field is neglected by assuming very small magnetic Reynolds number. Current density **J** including the Hall effect while neglecting thermoelectric and ion-slip effects is given by [11]

$$\begin{array}{c} J=\sigma \;[E+V\times B-\frac{1}{en_{e}}(J\times B)] \end{array}$$

where **J** is the current density, *σ* the electric conductivity of fluid, *e* the electric charge and *n*_*e*_ electron number density and *m* = *σB*_0_/*en*_*e*_ the Hall parameter. Here the electric field is considered to be *E* = 0. The wave geometry is considered as in [23]:

$$\begin{array}{ccc} {\bar{h}}_{1}\left(\bar{X},\bar{t}\right) & = & {\bar{d}}_{1}+{\bar{a}}_{1}\cos(\frac{2\pi }{\lambda }\;(\bar{X}-c\bar{t})),\;\;\;\;\;\;\;\;\;\;\;\;\;\;\;\;\;\;\;\;\;\;\;\;\;\;\;\;\;\;\;\;\;\;\;\;\;\text{upper}\;\text{wall,}\\ \bar{h}\left(\bar{X},\bar{t}\right) & = & {\bar{d}}_{2}+{\bar{b}}_{1}\cos(\frac{2\pi }{\lambda }\;(\bar{X}-c\bar{t})+\phi )\;\;\;\;\;\;\;\;\;\;\;\;\;\;\;\;\;\;\;\;\;\;\;\;\;\;\;\;\;\;\;\text{lower}\;\text{wall,} \end{array}$$ (1)

where ${\bar{a}}_{1,}{\bar{a}}_{2}$ are the wave amplitudes and the phase difference *ϕ* varies in the range 0 ≤ *ϕ* ≤ *π*. The case *ϕ* = 0 corresponds to the symmetric channel with waves out of phase and when *ϕ* = *π* the waves are in phase. Further *λ* is the wavelength, $\bar{t}$ the time and ${\bar{a}}_{1,}{\bar{a}}_{2},{\bar{d}}_{1},{\bar{d}}_{2}$ and *ϕ* satisfy ${\bar{a}}_{1}^{2}+{\bar{a}}_{2}^{2}+2\;{\bar{a}}_{1}{\bar{a}}_{2}\;\text{cos}\;\phi \leq {\left({\bar{d}}_{1}+{\bar{d}}_{2}\right)}^{2}$ in which *λ* is the wavelength, $\bar{t}$ the time and *b* the wave amplitude. Denoting the velocity components $\bar{U}$ and $\bar{V}$ along the $\bar{X}$ and $\bar{Y}$—directions respectively in the fixed frame, the velocity field **V** is

$$\begin{array}{c} V=[\bar{U}\left(\bar{X},\bar{Y},\bar{t}\right),\bar{V}\left(\bar{X},\bar{Y},\bar{t}\right),0]. \end{array}$$ (2)

The governing equations for the present flow problem are:

$$\begin{array}{c} \nabla \cdot V=0, \end{array}$$ (3)

$$\begin{array}{c} \rho \frac{dV}{dt}=\text{div}\;T+J\times B+\rho g\alpha \left(T-T_{0}\right), \end{array}$$ (4)

$$\begin{array}{c} \rho C_{p}\frac{dT}{dt}=\kappa \nabla^{2}T+T.L+Q. \end{array}$$ (5)

Here *ρ* is density, *C*_*p*_-the specific heat, *T*- the temperature, *κ*- the thermal conductivity, *Q*- the heat generation parameter and *d*/*dt*- the material derivative.

The expression of Cauchy stress tensor $\bar{\text{T}}$ [21] is

$$\begin{array}{c} \bar{T}=-p\bar{I}+\bar{S}, \end{array}$$ (6)

$$\begin{array}{c} \bar{S}+{\bar{\lambda }}_{1}{\bar{S}}^{\nabla }+\frac{1}{2}\;({\bar{\lambda }}_{1}-{\bar{\mu }}_{1})\;({\bar{A}}_{1}\bar{S}+\bar{S}\;{\bar{A}}_{1})=\mu {\bar{A}}_{1}, \end{array}$$ (7)

$$\begin{array}{c} {\bar{S}}^{\nabla }=\frac{d\bar{S}}{dt}-\bar{S}{\bar{L}}^{T}-\bar{L}\bar{S},\;\;\bar{L}=\text{grad}\;\bar{V}, \end{array}$$ (8)

in which $\bar{\text{I},}p,\bar{\text{S}},\mu ,{\bar{\text{S}}}^{\nabla }$ and ${\bar{\mu }}_{1}$ and ${\bar{\lambda }}_{1}$ respectively denote the identity tensor, the pressure, the extra stress tensor, the dynamic viscosity, the upper-convected derivative and the relaxation times. Introducing the transformations

$$\begin{array}{ccc} \bar{x} & = & \bar{X}-c\bar{t},\;\;\;\;\;\;\;\;\;\;\;\;\;\;\;\;\;\;\;\;\;\;\;\;\;\;\;\;\;\bar{y}=\bar{Y},\\ \bar{u}\left(\bar{x},\bar{y}\right) & = & \bar{U}-c,\;\;\;\;\;\;\;\;\;\;\;\;\;\;\;\;\;\;\;\;\;\;\;\;\;\;\;\;\;\;\;\;\bar{v}\left(\bar{x},\bar{y}\right)=\bar{V}, \end{array}$$ (9)

the equations in the wave frame become

$$\begin{array}{c} \frac{\partial \bar{u}}{\partial \bar{x}}+\frac{\partial \bar{v}}{\partial \bar{y}}=0, \end{array}$$ (10)

$$\begin{array}{c} \rho \;(\bar{u}\frac{\partial }{\partial \bar{x}}+\bar{v}\frac{\partial }{\partial \bar{y}})\;\bar{u}+\frac{\partial \bar{p}}{\partial \bar{x}}=\frac{\partial {\bar{S}}_{xx}}{\partial \bar{x}}+\frac{\partial {\bar{S}}_{xy}}{\partial \bar{y}}+\rho g\alpha \left(T-T_{0}\right)+\frac{\sigma B_{0}^{2}}{1+m^{2}}(m\bar{v}-\bar{u}-1), \end{array}$$ (11)

$$\begin{array}{c} \rho \;(\bar{u}\frac{\partial }{\partial \bar{x}}+\bar{v}\frac{\partial }{\partial \bar{y}})\;\bar{v}+\frac{\partial \bar{p}}{\partial \bar{y}}=\frac{\partial {\bar{S}}_{yx}}{\partial \bar{x}}+\frac{\partial {\bar{S}}_{yy}}{\partial \bar{y}}-\frac{\sigma B_{0}^{2}}{1+m^{2}}(m\bar{u}+\bar{v}+m), \end{array}$$ (12)

$$\begin{array}{c} \rho C_{p}\;[\bar{u}\frac{\partial }{\partial \bar{x}}+\bar{v}\frac{\partial }{\partial \bar{y}}]\;\bar{T}=\kappa \;[\frac{\partial^{2}\bar{T}}{\partial {\bar{x}}^{2}}+\frac{\partial^{2}\bar{T}}{\partial {\bar{y}}^{2}}]+{\bar{S}}_{xx}\frac{\partial \bar{u}}{\partial \bar{x}}+{\bar{S}}_{yy}\frac{\partial \bar{v}}{\partial y}+{\bar{S}}_{xy}\;[\frac{\partial \bar{v}}{\partial \bar{x}}+\frac{\partial \bar{u}}{\partial y}]+Q \end{array}$$ (13)

and upcoming Eqs (14)–(16) and (23)–(25) reproduces information already reported in detail in [22].

$$\begin{array}{ccc} 2\mu \frac{\partial \bar{v}}{\partial \bar{y}} & = & {\bar{S}}_{yy}+{\bar{\lambda }}_{1}\;(\bar{u}\frac{\partial {\bar{S}}_{yy}}{\partial \bar{x}}+\bar{v}\frac{\partial {\bar{S}}_{yy}}{\partial \bar{y}}-2\frac{\partial \bar{v}}{\partial \bar{y}}{\bar{S}}_{yy}-2\frac{\partial \bar{v}}{\partial \bar{x}}{\bar{S}}_{xy})+\frac{1}{2}\;({\bar{\lambda }}_{1}-{\bar{\mu }}_{1})\\ & & \times (4{\bar{S}}_{yy}\frac{\partial \bar{v}}{\partial \bar{y}}+2{\bar{S}}_{xy}\;(\frac{\partial u}{\partial y}+\frac{\partial v}{\partial x})), \end{array}$$ (14)

$$\begin{array}{ccc} \mu \;(\frac{\partial \bar{u}}{\partial \bar{y}}+\frac{\partial \bar{v}}{\partial \bar{x}}) & = & {\bar{S}}_{xy}+{\bar{\lambda }}_{1}\;(\bar{u}\frac{\partial {\bar{S}}_{xy}}{\partial \bar{x}}+\bar{v}\frac{\partial {\bar{S}}_{xy}}{\partial \bar{y}}+\frac{\partial \bar{v}}{\partial \bar{x}}{\bar{S}}_{xx}-\frac{\partial \bar{u}}{\partial \bar{y}}{\bar{S}}_{yy})+\frac{1}{2}\;({\bar{\lambda }}_{1}-{\bar{\mu }}_{1})\\ & & \times (({\bar{S}}_{xx}+{\bar{S}}_{xy})\;(\frac{\partial \bar{u}}{\partial \bar{y}}+\frac{\partial \bar{v}}{\partial \bar{x}})), \end{array}$$ (15)

$$\begin{array}{ccc} 2\mu \frac{\partial \bar{u}}{\partial \bar{x}} & = & {\bar{S}}_{xx}+{\bar{\lambda }}_{1}\;(\bar{u}\frac{\partial {\bar{S}}_{xx}}{\partial \bar{x}}+\bar{v}\frac{\partial {\bar{S}}_{xx}}{\partial \bar{y}}-2\frac{\partial \bar{u}}{\partial \bar{x}}{\bar{S}}_{xx}-2\frac{\partial \bar{u}}{\partial \bar{y}}{\bar{S}}_{xy})+\frac{1}{2}\;({\bar{\lambda }}_{1}-{\bar{\mu }}_{1})\\ & & \times (4{\bar{S}}_{xx}\frac{\partial \bar{u}}{\partial \bar{x}}+2{\bar{S}}_{xy}\;(\frac{\partial u}{\partial y}+\frac{\partial v}{\partial x})), \end{array}$$ (16)

where $\bar{u}$ and $\bar{v}$ are the velocity components along the $\bar{x}$ and $\bar{y}$ directions, respectively.

Denoting *Ec*, Pr, *δ*, *Re* and *M* as the Eckert, Prandtl, wave, Reynolds, and Hartman numbers respectively; *α* the amplitude ratio, *m* the hall current parameter, *E* the electric field strength, *γ* the non-dimensional temperature, *ψ* the stream function, *λ*_1_ and *μ*_1_ the non-dimensional relaxation times, and *T*_0_ and *T*_1_ the dimensional temperature at walls and defining

$$\begin{array}{ccc} \lambda_{1} & = & \frac{{\bar{\lambda }}_{1}c}{a},\;x=\frac{\bar{x}}{\lambda },\;y=\frac{\bar{y}}{a},\;t=\frac{c\bar{t}}{\lambda },\;p=\frac{a^{2}\bar{p}}{c\lambda \mu },\;M=\frac{\sigma B_{0}^{2}d_{1}}{\mu },\;\;u=\frac{\bar{u}}{c},\;v=\frac{\bar{v}}{c},\\ \delta & = & \frac{a}{\lambda },\;{\bar{S}}_{ij}=\frac{a{\bar{S}}_{ij}}{\mu c}\;(\;\text{for}\;\;\;i,j\;=1,2,3),\;\mu_{1}=\frac{{\bar{\mu }}_{1}c}{a},\;\beta =\frac{Qd_{1}^{2}}{k(T_{1}-T_{0})}\;\;\gamma =\frac{\bar{T}-T_{0}}{T_{1}-T_{0}},\\ Ec & = & \frac{c^{2}}{C_{p}T_{0}},\;\mathrm{Pr}=\frac{\mu C_{p}}{\kappa },\;b=\frac{{\bar{b}}_{1}}{{\bar{d}}_{1}},\;h_{1}=\frac{{\bar{h}}_{1}}{{\bar{d}}_{1}},\;h_{2}=\frac{{\bar{h}}_{2}}{{\bar{d}}_{1}},\;a=\frac{{\bar{a}}_{1}}{{\bar{d}}_{1}},\;Re=\frac{ca\rho }{\mu }. \end{array}$$ (17)

The wall surfaces in dimensionless variables are expressed as

$$\begin{array}{ccc} h_{1} & = & 1+a\cos(2\pi x),\\ h_{2} & = & -d-b\cos(2\pi x+\phi ). \end{array}$$ (18)

Introducing

$$\begin{array}{c} u=\frac{\partial \Psi }{\partial y}\;,v=-\delta \frac{\partial \Psi }{\partial x} \end{array}$$ (19)

continuity Eq (10) is identically satisfied and Eqs (11)–(16) yield

$$\begin{array}{c} Re\delta \;(\frac{\partial \Psi }{\partial y}\frac{\partial }{\partial x}-\frac{\partial \Psi }{\partial x}\frac{\partial }{\partial y})\;\frac{\partial \Psi }{\partial y}+\frac{\partial p}{\partial x}=\delta \frac{\partial S_{xx}}{\partial x}+\frac{\partial S_{xy}}{\partial y}-\frac{M}{m^{2}+1}(u+1)+Gr\;\gamma , \end{array}$$ (20)

$$\begin{array}{c} -Re\delta^{2}\;(\frac{\partial \Psi }{\partial y}\frac{\partial }{\partial x}-\frac{\partial \Psi }{\partial x}\frac{\partial }{\partial y})\;\frac{\partial \Psi }{\partial x}+\frac{\partial p}{\partial y}=\delta \;(\delta \frac{\partial S_{yx}}{\partial x}+\frac{\partial S_{yy}}{\partial y})-\delta^{2}\frac{M}{m^{2}+1}(v), \end{array}$$ (21)

$$\begin{array}{ccc} & & \delta \mathrm{Pr}\text{Re}\;[\frac{\partial \Psi }{\partial y}\frac{\partial \gamma }{\partial x}-\frac{\partial \Psi }{\partial x}\frac{\partial \gamma }{\partial y}]=\delta^{2}\frac{\partial^{2}\gamma }{\partial x^{2}}+\frac{\partial^{2}\gamma }{\partial y^{2}}+\beta +Ec\mathrm{Pr}\times \\ & & \;\;\;\;\;\;\;\;\;\;\;\;\;\;\;\;\;\;\;\;\;\;\;\;\;\;\;\;\;\;\;\;\;\;\;\;\;\;\;\;\;\;\;\;\;\;[\delta \left(S_{xx}-S_{yy}\right)\frac{\partial^{2}\Psi }{\partial x\partial y}+\left(\frac{\partial^{2}\Psi }{\partial y^{2}}-\delta^{2}\frac{\partial^{2}\Psi }{\partial x^{2}}\right)S_{xy}], \end{array}$$ (22)

$$\begin{array}{ccc} 2\delta \frac{\partial^{2}\Psi }{\partial y\partial x} & = & S_{xx}+\lambda_{1}\;[(\delta \frac{\partial \Psi }{\partial y}\frac{\partial }{\partial x}+\frac{\partial \Psi }{\partial x}\frac{\partial }{\partial y})\;S_{xx}-2\;(\delta \frac{\partial^{2}\Psi }{\partial x\partial y}S_{xx}+\frac{\partial^{2}\Psi }{\partial y^{2}}S_{xy})]\\ & & +\frac{1}{2}\;(\lambda_{1}-\mu_{1})\;(4\delta S_{xx}\frac{\partial^{2}\Psi }{\partial x\partial y}+2S_{xy}\;(\frac{\partial^{2}\Psi }{\partial y^{2}}-\delta^{2}\frac{\partial^{2}\Psi }{\partial x^{2}})), \end{array}$$ (23)

$$\begin{array}{ccc} -2\delta \frac{\partial^{2}\Psi }{\partial y\partial x} & = & S_{yy}+\lambda_{1}\;[\delta \;(\frac{\partial \Psi }{\partial y}\frac{\partial }{\partial x}-\frac{\partial \Psi }{\partial x}\frac{\partial }{\partial y})\;S_{yy}+2\delta \;(\delta \frac{\partial^{2}\Psi }{\partial x^{2}}S_{xy}+\frac{\partial^{2}\Psi }{\partial x\partial y}S_{yy})]\\ & & +\frac{1}{2}\;(\lambda_{1}-\mu_{1})\;[2S_{xy}\;(\frac{\partial^{2}\Psi }{\partial y^{2}}-\delta^{2}\frac{\partial^{2}\Psi }{\partial x^{2}})-4\delta S_{yy}\frac{\partial^{2}\Psi }{\partial x\partial y}], \end{array}$$ (24)

$$\begin{array}{ccc} \frac{\partial^{2}\Psi }{\partial y^{2}}-\delta^{2}\frac{\partial^{2}\Psi }{\partial x^{2}} & = & S_{xy}+\lambda_{1}\;[\delta \;(\frac{\partial \Psi }{\partial y}\frac{\partial }{\partial x}-\frac{\partial \Psi }{\partial x}\frac{\partial }{\partial y})\;S_{xy}-(\frac{\partial^{2}\Psi }{\partial y^{2}}S_{yy}-\delta^{2}\frac{\partial^{2}\Psi }{\partial x^{2}}S_{xx})]\\ & & +\frac{1}{2}\;(\lambda_{1}-\mu_{1})\;[(S_{xx}+S_{yy})\;(\frac{\partial^{2}\Psi }{\partial y^{2}}-\delta^{2}\frac{\partial^{2}\Psi }{\partial x^{2}})]. \end{array}$$ (25)

Under the assumptions of long wavelength and low Reynolds number we obtain

$$\begin{array}{c} \frac{\partial p}{\partial x}=\frac{\partial S_{xy}}{\partial y}-\frac{M}{m^{2}+1}\;(\frac{\partial \Psi }{\partial y}+1)+Gr\;\gamma , \end{array}$$ (26)

$$\begin{array}{c} \frac{\partial p}{\partial y}=0, \end{array}$$ (27)

$$\begin{array}{c} \frac{\partial^{2}\gamma }{\partial y^{2}}+Br\frac{\partial^{2}\Psi }{\partial y^{2}}S_{xy}+\beta =0, \end{array}$$ (28)

$$\begin{array}{c} S_{xx}=(\lambda_{1}+\mu_{1})\;\frac{\partial^{2}\Psi }{\partial y^{2}}S_{xy}, \end{array}$$ (29)

$$\begin{array}{c} S_{yy}=(-\lambda_{1}+\mu_{1})\;S_{xy}\frac{\partial^{2}\Psi }{\partial y^{2}}, \end{array}$$ (30)

$$\begin{array}{c} S_{xy}=\frac{\Psi_{yy}}{1+\xi \Psi_{yy}^{2}}, \end{array}$$ (31)

with $\left(\lambda_{1}^{2}-\mu_{1}^{2}\right)=\zeta .$

The dimensionless boundary conditions in wave frame are

$$\begin{array}{ccc} \Psi & = & \frac{F}{2},\;\;\;\frac{\partial \Psi }{\partial y}=-1,\;\;\;\;\;\;\;\;\;\;\;\gamma =0\;\;\;\;\;\;\;\;at\;\;\;\;y=h_{1},\\ \Psi & = & -\frac{F}{2},\;\;\;\frac{\partial \Psi }{\partial y}=-1,\;\;\;\;\;\;\;\;\;\;\gamma =1\;\;\;\;\;\;\;\;at\;\;\;\;y=h_{2} \end{array}$$ (32)

where *F* is the dimensionless time mean flow rate in the wave frame. If *θ* is the dimensionless time mean flow rate in the laboratory frame then *θ* = *F* + 1 + *d* where

$$\begin{array}{c} F=\int_{h_{2}}^{h_{1}}\frac{\partial \Psi }{\partial y}\;dy. \end{array}$$ (33)

Employing Eqs (26) and (31) one obtains

$$\begin{array}{c} \frac{\partial p}{\partial x}=\frac{\partial }{\partial y}\;[\Psi_{yy}\{1-\xi \;{\left(\frac{\partial^{2}\Psi }{\partial y^{2}}\right)}^{2}\}]-\frac{M}{m^{2}+1}\;(\frac{\partial \Psi }{\partial y}+1)+Gr\;\gamma , \end{array}$$ (34)

Eqs (27) and (34) yield

$$\begin{array}{c} \frac{\partial^{4}\Psi }{\partial y^{4}}-\xi \frac{\partial^{2}}{\partial y^{2}}{\left(\frac{\partial^{2}\Psi }{\partial y^{2}}\right)}^{3}-\frac{M}{m^{2}+1}\frac{\partial^{2}\Psi }{\partial y^{2}}+Gr\;\frac{\partial \gamma }{\partial y}=0. \end{array}$$ (35)

The pressure rise Δ*P*_*λ*_ is written as follows:

$$\begin{array}{c} \Delta P_{\lambda }=\int_{0}^{1}\frac{dp}{dx}dx, \end{array}$$ (36)

The above coupled ordinary differential Eqs (26)–(28) subject to boundary conditions Eq (32) are solved for stream function, temperature distribution and pressure gradient component by utilizing built-in routine for solving non-linear ordinary differential equation by shooting method using symbolic computational software maple.

## 3 Results and Discussion

**Fig 1:**
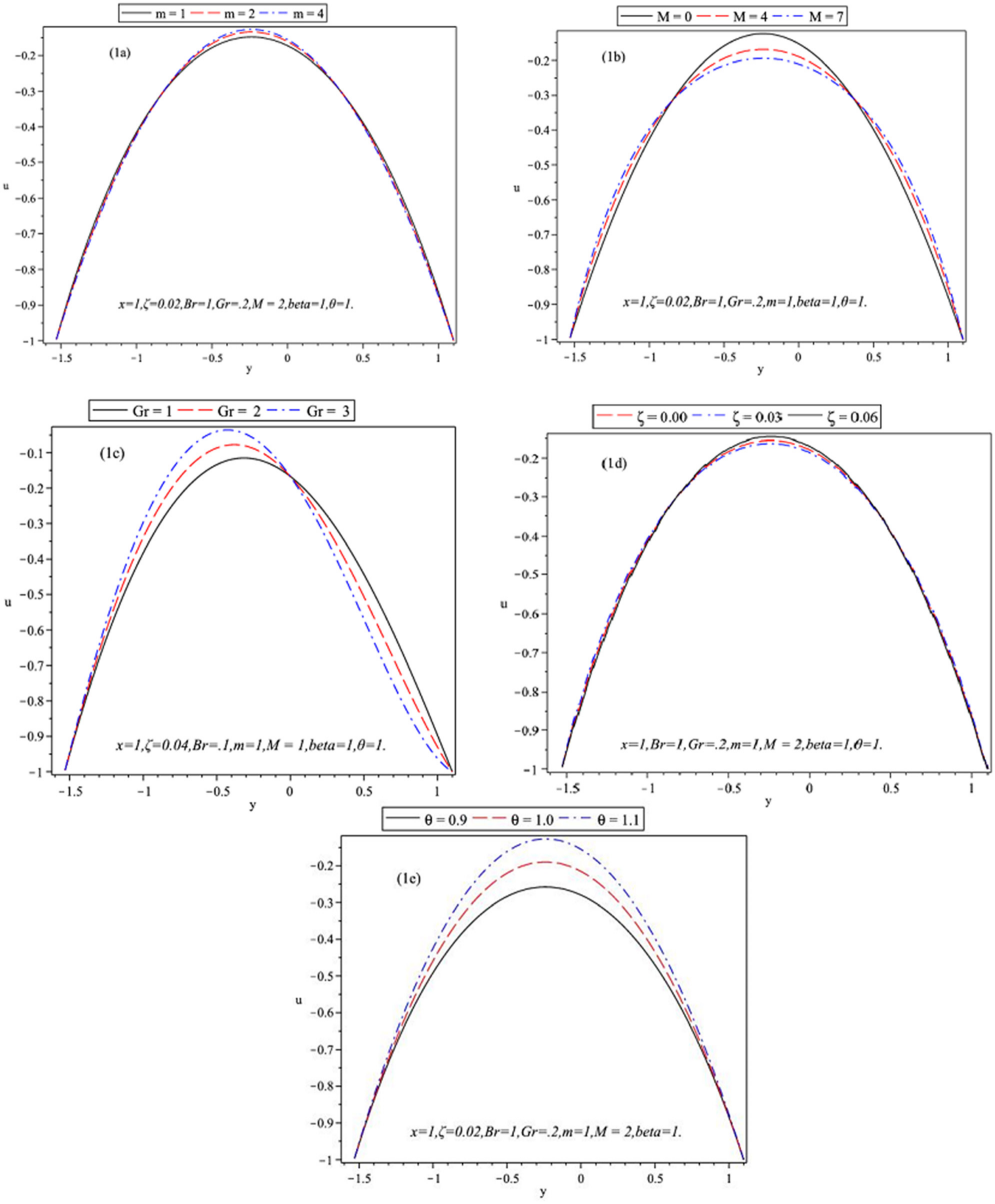
Influence of *u* versus *y* for *b* = 0.5, *a* = 0.1, *d* = 1.1, *ϕ* = *π*/6.

**Fig 2:**
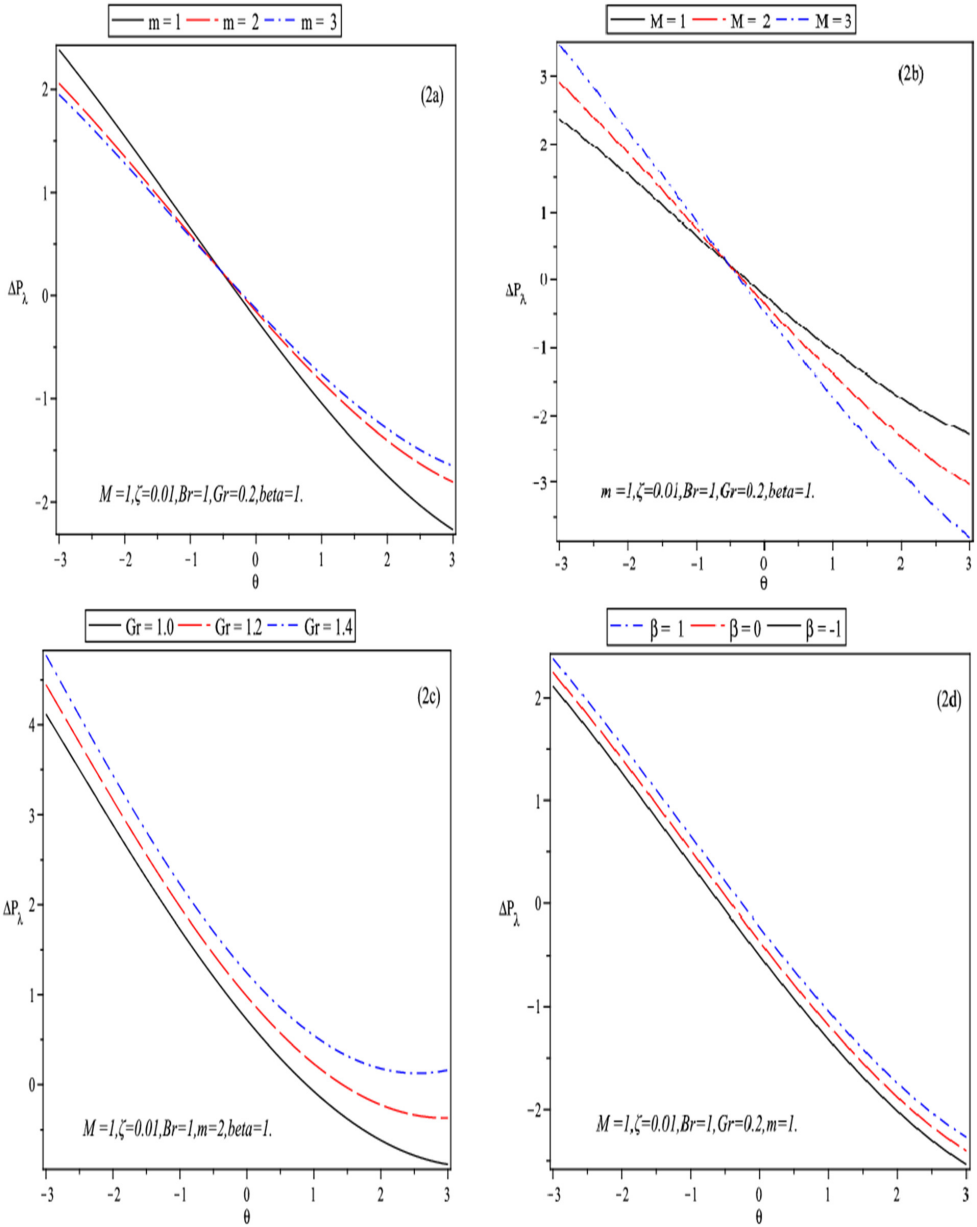
Inflence of Δ*P*_*λ*_ versus *θ* for *b* = 0.5, *a* = 0.1, *d* = 1.1, *ϕ* = *π*/6.

**Fig 3:**
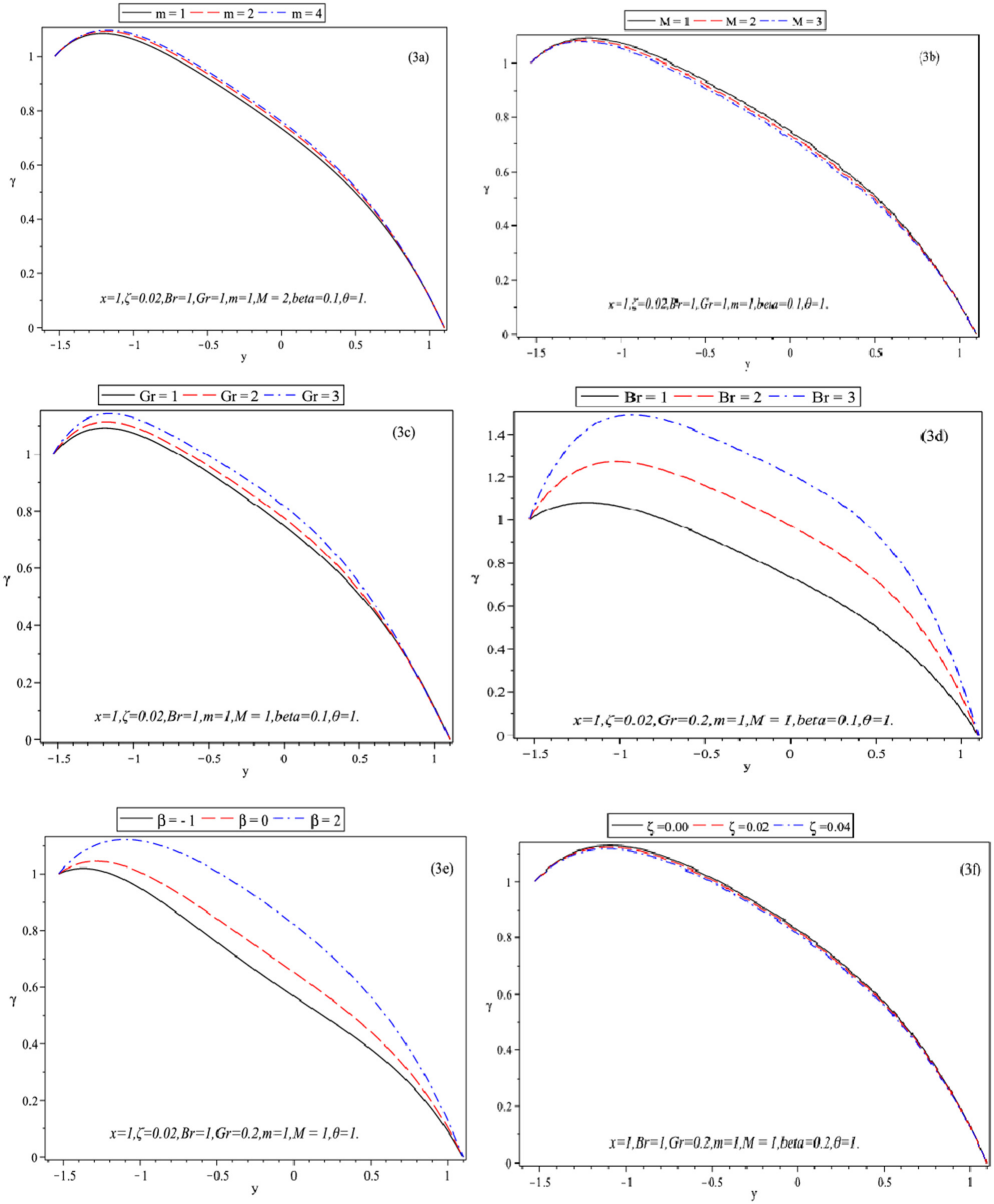
Plots showing Temperature *γ* versus *y*.

In a rectangular system, Magnetohydrodynamic pseudoplastic flow subject to heat generation and Hall current is studied. Magnetic fields are safe and have healing benefits in human body. Their actions on physiological processes include: ion movement, changes in electrical potentials, chemical reactions, effects on proteins, hormones, starches, lipids and cellular processes. Applied magnetic field can produce required intensity in the target tissue say abdomen, kidney. The Magnetic field needs to be much stronger to reach and affect the kidneys. The Hall effect also becomes important in an ionized medium. The influence of five important parameters *Br*, *ξ*, *m*, *M* and *Gr* (Brinkman number, relaxation time, Hall parameter, Hartman number, and Grashof number) on *u*, Δ*P*_*λ*_ and *γ* (the velocity distribution pressure rise and temperature) is represented graphically in the Figs 1–3.

The variations of *m*, *M*, *Gr*, *ξ* and *θ* on the velocity have been plotted in Fig 1(a)–1(e) by fixing values of *b* = 0.5, *a* = 0.1, *d* = 1.1 and *ϕ* = *π*/6. Hall current plays an important role in determining features of channel flow. Fig 1a shows that velocity profile decreases with an increase in the value of Hall parameter *m*. Hall effect is negligible if the applied magnetic field is small. However, the case is reversed for strong magnetic fields. It produces an electric current which generates transverse fluid motion, after interacting with magnetic field. This is how it affects channel flow and has applications in all small channels in human body. Hall meters are the most significant in bioengineering systems. The sensitivity of these devices can determine the high and low-end intensities to be detected. Fig 1e reports an established result i.e. velocity increases with an increase in flow rate. Fig 1b and 1d show that velocity near the channel wall is not similar to the centre of channel and decreases with an increase in *M* and *ξ* at *y* = 0. Magnetic field tend to retard the fluid flow in axial direction. Magnetic field therapy with Hall effects is used in life support devices intended to be implanted in human body to support, sustain and protect human life. In Fig 1c it is seen that the magnitude of velocity field increases in the region 0 ≤ *y* ≤ 1.1 and it decreases in the region −1.6 ≤ *y* ≤ 0 with an increase in *Gr*.

The pressure rise versus volume flow rate is drawn in Fig 2(a)–2(d). It is clear from Fig 2a that in pumping region (Δ*P*_*λ*_ > 0) the pumping rate increases by increasing Hall current parameter *m*. While in the co- pumping region Δ*P*_*λ*_ < 0 and free pumping Δ*P*_*λ*_ = 0, the pumping rate increases by increasing *m*. The situation is reversed in Fig 2b, where an increase in *M* leads to an increase in the pressure rise in pumping region (Δ*P*_*λ*_ > 0). Moving ions induce currents in the tissue or medium. This interaction serves as basis of magnetically-induced blood flow. It is also observed that Δ*p*_*λ*_ is an increasing function of local Grashof number *Gr* and heat generation parameter *β*.

Fig 3(a)–3(f) elucidates the temperature distribution *γ* of fluid for different parameters. It is observed from Fig 3a and 3b that the value of temperature distribution *γ* increases when *m* increases. However, the absolute value of temperature distribution *γ* decreases upon increasing *M*. Brinkman number describes viscous heating effect and is used to determine the relationship between the heat produced by dissipation and exchanged at the wall. Grashof number studies the domination of convection modes (free or forced) in channel flow. Positive values of Brinkman number represents lower fluid temperature than wall in case of uniform temperature conditions. The effect of *Br* and *Gr* on distribution *γ* have been described in Fig 3c and 3d. Obviously, the magnitude of *γ* is an increasing function of *Br* and *Gr*. Fig 3e plots the variations of *γ* against *y* for different values of heat sink source parameter *β* (heat sink (*β* = −1), no heat generation (*β* = 0), heat source (*β* = +1)). It is noticed that magnitude of *γ* increases when *β* is increased. Damaged tissues are more metabolically active. Here enhanced heat transport (creating source or sink) can facilitate more rapid healing. Lower temperatures preserve living tissue. This technique is often applied during surgeries with patients who experience head trauma. *ζ* is responsible for fluid elasticity and shear thinning behavior. The influence of pseudoplastic fluid parameter *ζ* on *γ* is opposite to *β* (Fig 3f). The present study is important to have a basic understanding of treating human tissues and organs with stones by utilizing the Hall current, heat convection and heat generation.

## 4 Conclusions

Present study theoretically examines the influence of Hall current, heat convection and heat generation on peristaltic transport of pseudoplastic fluid. We specifically note the following results:

1. Hall current reduces the axial velocity *u* in pseudoplastic fluid.
2. The velocity distribution has opposite behavior near walls *h*_1_ and *h*_2_ under increase in *Gr*. Pumping rate increases with an increase in *M* and decreases with *ξ*.
3. Magnitude of velocity and temperature is larger for pseudoplastic fluid as compared to viscous fluid.
4. Pressure rise per wavelength decreases with Hall parameter *m* and increases in all pumping regions with *β* and *Gr*.
5. Heat generation parameter increases temperature distribution *γ*.
6. Temperature distribution is directly proportional to *Br* and *Gr*.
